# Oral Health Literacy and Its Association With Oral Health Status Among Patients Attending a Tertiary Dental Hospital: A Cross-Sectional Study

**DOI:** 10.7759/cureus.115884

**Published:** 2026-09-07

**Authors:** Shivani Gupta, Sumit Garg, Rajesh K Gupta, Preety Gupta, Priya Gupta

**Affiliations:** 1 Pediatric Dentistry, National Dental College Derabassi, Dera Bassi, IND; 2 Periodontics, Swami Devi Dyal Hospital and Dental College, Panchkula, IND; 3 Oral and Maxillofacial Radiology, Swami Devi Dyal Hospital and Dental College, Panchkula, IND; 4 Public Health Dentistry, Swami Devi Dyal Hospital and Dental College, Panchkula, IND; 5 Oral Pathology, Luxmi Bai Institute of Dental Sciences and Hospital, Patiala, IND

**Keywords:** dental caries, health education, oral health literacy, oral hygiene, periodontal disease, tooth loss

## Abstract

Introduction

Limited oral health literacy (OHL) can adversely affect preventive behaviors, dental service utilization, treatment adherence, and oral health outcomes. Evidence linking literacy to clinically assessed oral health outcomes among patients attending tertiary dental hospitals remains limited.

Objective

To assess OHL and determine its association with clinically assessed oral health conditions among adult dental patients and to identify sociodemographic, behavioral, and healthcare-related factors associated with low to moderate OHL.

Materials and methods

A hospital-based, cross-sectional analytical study was conducted among 423 adults attending a tertiary dental hospital. The 17-item Oral Health Literacy-Adult Questionnaire (OHL-AQ) was used to assess OHL. Oral mucosal lesions, tooth loss, oral hygiene, periodontal pocketing, and dental caries were clinically evaluated. Associations were examined using bivariate tests and multivariable regression.

Results

Low, moderate, and high OHL were found in 31.0%, 15.6%, and 53.4% of participants, respectively. The prevalence of dental caries, periodontal pocketing, tooth loss, and poor oral hygiene was 57.4%, 41.4%, 43.7%, and 32.9%, respectively. After adjustment, low OHL was independently associated with a higher prevalence of dental caries (aPR, 1.46; 95% CI, 1.22-1.66), periodontal pocketing (aPR, 1.73; 95% CI, 1.32-2.12), tooth loss (aPR, 1.75; 95% CI, 1.38-2.09), and poor oral hygiene (aPR, 1.80; 95% CI, 1.31-2.32). Secondary education or below, rural residence, tobacco use, infrequent brushing, and irregular preventive dental visits were independently associated with low-to-moderate OHL.

Conclusions

Limited OHL was associated with unfavorable behaviors and a greater burden of adverse oral health outcomes. Because these findings are cross-sectional and no intervention was tested, literacy-sensitive communication and preventive strategies warrant further evaluation as potential means of improving oral health outcomes in this population.

## Introduction

Oral health literacy (OHL) is the capacity to obtain, understand, appraise, and apply oral health information in order to make decisions about prevention, treatment, and the use of dental services [1]. It extends beyond reading ability to encompass functional literacy, communication, numeracy, critical appraisal of health information, and the ability to follow preventive and treatment instructions [2]. Adequate OHL is clinically important because patients must understand medication schedules, consent information, postoperative instructions, dietary advice, oral hygiene guidance, treatment options, and follow-up requirements [3]. When OHL is limited, dentist-patient communication becomes more difficult; this can reduce adherence to treatment goals, promote symptom-driven rather than preventive attendance, and contribute to delayed diagnosis and avoidable complications [4].

Oral diseases are a major public health problem that is largely preventable [5]. They are estimated to affect approximately 3.7 billion people worldwide, and untreated dental caries in permanent teeth is the single most common health condition reported in the Global Burden of Disease 2021 study [6]. Dental caries, periodontal disease, tooth loss, and oral cancer can cause pain, infection, difficulty chewing, impaired communication, reduced productivity, and diminished social functioning and quality of life. These conditions disproportionately affect socially and economically disadvantaged groups, including people with inadequate fluoride exposure, high sugar intake, tobacco or alcohol use, limited access to preventive services, and low educational attainment [5].

Such disparities suggest that access to clinical services alone may be insufficient unless patients can comprehend and act on oral health information [7]. Prior work indicates that OHL can influence oral health behaviors, service utilization, and clinical outcomes [1]. Low OHL has been linked to reduced self-efficacy and perceived oral health, greater dental neglect, more dental plaque, emergency and pain-driven visits, missing teeth, poorer periodontal health, and a greater impact of oral conditions on quality of life [8]. The evidence is not entirely consistent, however, because studies differ in the literacy instruments used, the outcomes defined, the populations sampled, and the analytical methods applied [3]. Many earlier studies relied on self-reported measures or word-recognition tests, included limited clinical assessment, or did not adjust for relevant sociodemographic and behavioral factors [4]. Previous Indian studies have examined OHL among adults attending dental institutions and have reported associations with clinical oral health outcomes, including dental caries, periodontal status, and tooth loss [9,10]. More broadly, OHL has been associated with oral health behaviors and clinical outcomes in adult populations [4,8]. However, these studies have differed in the OHL instruments used and in the clinical outcomes assessed [9,10].

Despite increasing recognition of the importance of OHL, evidence linking OHL with multiple clinically assessed oral health outcomes remains limited and heterogeneous. Existing studies have differed in the populations studied, instruments used to measure OHL, clinical outcomes assessed, and approaches to controlling for potential confounding factors [3,4]. Evidence from India is also relatively limited, with available studies conducted in different populations and using varying measures of oral health status [11,12]. Therefore, further evaluation of OHL alongside standardized clinical assessment of dental caries, periodontal disease, oral hygiene, tooth loss, and oral mucosal conditions is warranted. Such evidence may help identify patients who could benefit from literacy-sensitive communication, preventive counselling, and improved utilization of dental services. This need is particularly relevant in tertiary dental care settings, where patients may have substantial oral health needs and diverse sociodemographic and educational backgrounds.

The primary objective of this study was to assess the level of OHL and determine its association with clinically assessed oral health conditions among adult patients attending a tertiary dental hospital. The secondary objectives were to describe the prevalence of major oral health conditions, including dental caries, periodontal pocketing, tooth loss, poor oral hygiene, and oral mucosal lesions, and to identify sociodemographic, behavioral, and healthcare-related factors associated with low to moderate OHL.

## Materials and methods

Study design and setting

A hospital-based, cross-sectional analytical study was conducted among adult patients attending the outpatient Department of Oral Medicine and Radiology, Swami Devi Dyal Hospital and Dental College, Barwala, Panchkula, Haryana, India, between June 2024 and April 2025.

Study Population and Sampling

The sampling frame comprised all consecutive adult patients aged 18 years or older who attended the outpatient Department of Oral Medicine and Radiology of the study hospital during the study period (June 2024 to April 2025) and met the predefined eligibility criteria. Consecutive sampling was used, whereby eligible patients presenting during the study period were approached for participation until the required sample size was achieved. Patients requiring emergency treatment or those with severe systemic illness, cognitive impairment, or communication difficulties were excluded. An age threshold of 18 years was used because the study focused on adult OHL, oral health behaviors, healthcare utilization, and clinically assessed oral health conditions in an adult dental care population.

Sample-Size Calculation

The sample size was calculated primarily to estimate the prevalence of low-to-moderate OHL with 95% confidence and 5% precision. This prevalence-based calculation was not intended to establish statistical power for the subsequent multivariable regression analyses involving multiple covariates and clinical outcomes. These regression analyses were considered exploratory/analytical secondary analyses. The initial calculated sample size was 384 participants; after allowing for a 10% nonresponse rate, the final required sample size was 423 participants.

The sample size was calculated using the formula:

 n = Z²p(1 - p)/d²

Using Z = 1.96, p = 0.50, and d = 0.05 gives approximately 384 participants. Adding 10% for nonresponse: 384 × 1.10 = 422.4 ≈ 423 participants.

The approach was consistent with previous cross-sectional studies evaluating OHL among dental care populations in India [11].

Data-Collection Instrument

A structured questionnaire was used to collect sociodemographic data, oral health behaviors, tobacco and alcohol use, dietary habits, and dental service utilization. OHL was assessed using the 17-item Oral Health Literacy-Adult Questionnaire (OHL-AQ), which evaluates reading comprehension, numeracy, listening, and decision-making [13]. The questionnaire was administered in English, and all participants were proficient in English and were able to complete the questionnaire without assistance. Participants were required to be able to read and understand English sufficiently to complete the questionnaire independently because the validated English version of the OHL-AQ was used. English proficiency was assessed pragmatically based on the participant’s ability to understand the study information and complete the questionnaire without assistance; no formal language or literacy proficiency test was administered. The OHL-AQ has established psychometric properties, including content and face validity, internal consistency, and test-retest reliability [13]. The questionnaire was pilot tested for clarity and feasibility among 20 participants, who were excluded from the final analysis. Each correct response was assigned one point, yielding a maximum score of 17. Scores of 0-9, 10-11, and 12-17 were classified as low, moderate, and high OHL, respectively. The OHL-AQ was used with reference to the original published instrument [13]. The complete questionnaire is not reproduced because of copyright restrictions.

Clinical Oral Examination

A single trained examiner conducted all clinical examinations under standardized conditions using a plane mouth mirror and a Community Periodontal Index (CPI) probe under adequate illumination. All examinations were performed under standardized clinical conditions using the same examination sequence and instruments throughout the study. The clinical indices were recorded according to their respective standard scoring criteria, and the predefined diagnostic thresholds were applied consistently to classify participants. Dental caries experience was recorded using the World Health Organization Decayed, Missing, and Filled Teeth (DMFT) index; a participant was classified as having dental caries when one or more teeth showed untreated coronal decay (D component ≥ 1). Periodontal pocketing was assessed using the CPI, with a highest sextant CPI score of ≥3 used to indicate the presence of periodontal pockets. Oral hygiene was assessed using the Simplified Oral Hygiene Index (OHI-S), and a score >3.0 was classified as poor oral hygiene. Lesions were identified and classified based on clinical examination according to these criteria. Histopathological confirmation was not routinely performed as part of the study. Tooth loss was defined as the loss of one or more permanent teeth for any reason, excluding third molars. To standardize examination and scoring, the examiner underwent training and calibration before data collection; however, a formal intra-examiner reliability statistic was not calculated.

Study Variables and Quality Control

The primary exposure was OHL. The principal outcomes were dental caries, periodontal pocketing, poor oral hygiene, tooth loss, and oral mucosal lesions. Sociodemographic variables included age, sex, residence, educational attainment, and household income. Behavioral variables included current tobacco use, daily sugary food intake, and tooth brushing frequency. Healthcare utilization variables included regular preventive dental visits. These variables were categorized later in the Results section and were considered based on their potential relevance to OHL and oral health conditions. Completed forms were checked daily, coded anonymously, and verified before analysis.

Statistical analysis

Data were analyzed using IBM SPSS Statistics version 27. Analysis syntax is not publicly available. Categorical variables were compared using the chi-square test or Fisher’s exact test, as appropriate, while continuous variables were compared using the independent-samples t-test or one-way ANOVA, as appropriate.

Potential covariates were selected a priori based on clinical and biological plausibility and on factors previously reported in the literature to be associated with OHL and oral health outcomes. For the analysis of factors associated with low-to-moderate OHL, the multivariable binary logistic regression model included age, sex, residence, education, monthly household income, current tobacco use, toothbrushing frequency, and regular preventive dental visits. These variables were specified a priori rather than selected solely on the basis of statistical significance in bivariate analyses.

For the association between OHL and clinical oral health outcomes, separate modified Poisson regression models with robust variance were adjusted for age, sex, education, monthly household income, and current tobacco use. Residence was not included in these primary models because it was not prespecified as a confounder of the OHL-clinical outcome associations. Toothbrushing frequency, preventive dental visits, and dietary sugar intake were not included in these primary outcome models because they may represent behavioral factors through which OHL could be associated with oral health outcomes. Given the cross-sectional design, these variables were not assumed to be established mediators. Multicollinearity among the independent variables was assessed using the variance inflation factor (VIF), with VIF values <5 considered indicative of acceptable levels of multicollinearity. A sensitivity analysis was also performed by repeating the multivariable model after excluding education because of its potentially strong association with OHL. For the analysis of oral health outcomes, separate multivariable modified Poisson regression models with robust variance were constructed and adjusted for age, sex, education, household income, and tobacco use, with high OHL serving as the reference category. Adjusted prevalence ratios (aPRs) with 95% CIs were reported, and a two-sided p-value <0.05 was considered statistically significant.

Ethical considerations

Ethical approval for the study was obtained from the Institutional Ethical Committee, Swami Devi Dyal Hospital and Dental College, Barwala, Panchkula, Haryana, India (approval date: 22 May 2024). The study was conducted in accordance with the principles of the Declaration of Helsinki and the ethical guidelines of the Indian Council of Medical Research (ICMR). The nature and purpose of the study were explained to all participants, and written informed consent was obtained from each participant before enrollment. Participation was voluntary, and confidentiality was maintained throughout.

## Results

Participant recruitment and response rate

A total of 468 patients attending the tertiary dental hospital were assessed for eligibility. Of these, 45 were excluded: 29 did not meet the inclusion criteria, and 16 declined to participate. The remaining 423 patients completed the sociodemographic questionnaire, the OHL assessment, and the standardized clinical examination and were included in the final analysis. All 423 participants were considered proficient in English and completed the English version of the OHL-AQ without assistance. The overall participation rate was 90.4% of all patients assessed and 96.4% of eligible patients invited. No participant in the final sample had sufficient missing questionnaire or clinical data to preclude analysis. The recruitment process is summarized in Figure 1.

**Figure 1:**
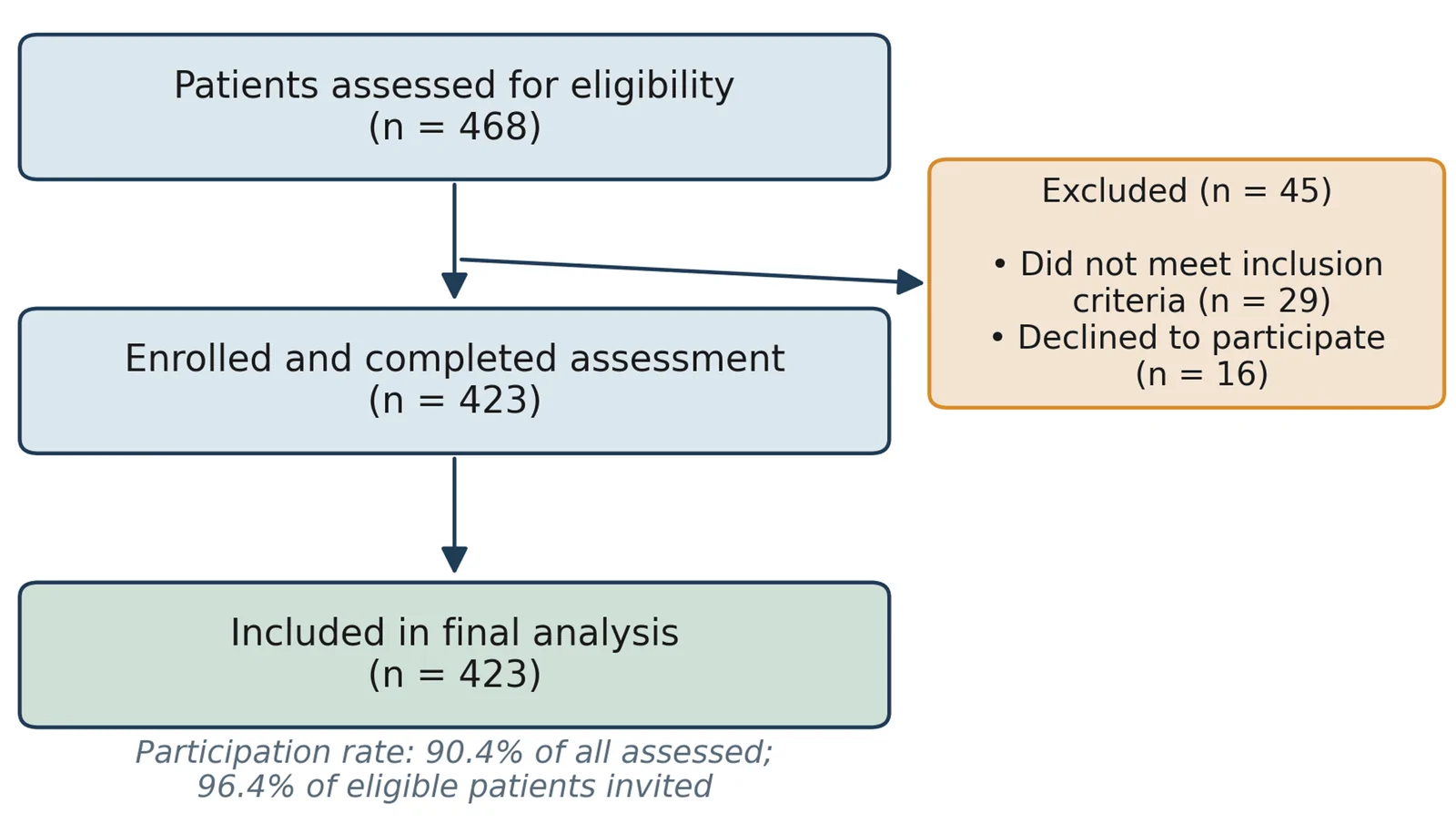
Flow diagram of participant recruitment, exclusions, and inclusion in the final analytical sample.

Sociodemographic and behavioral characteristics

Of the 423 participants, 231 (54.6%) were male and 192 (45.4%) were female. Most were aged 30-44 years (40.0%), followed by those aged 45-59 years (28.1%). Urban residents comprised 59.6% of the sample. Regarding education, 39.0% had completed secondary education, and 37.4% held a graduate or higher qualification. Middle-income and low-income participants accounted for 43.5% and 41.1%, respectively. Current tobacco use was reported by 27.7%, and 41.6% consumed sugary foods daily. Only 45.6% brushed at least twice daily, and 32.2% reported regular preventive dental visits. Detailed characteristics are presented in Table 1.

**Table 1:** Sociodemographic, socioeconomic, and oral health characteristics of participants (N = 423). Values are presented as n (%).

Characteristic	Category	n	%
Age group (years)	18-29	108	25.5
	30-44	169	40
	45-59	119	28.1
	≥60	27	6.4
Sex	Male	231	54.6
	Female	192	45.4
Residence	Urban	252	59.6
	Rural	171	40.4
Education	No formal education/primary	100	23.6
	Secondary	165	39
	Graduate or higher	158	37.4
Employment	Employed	129	30.5
	Self-employed	92	21.7
	Homemaker	98	23.2
	Student	42	9.9
	Unemployed/retired	62	14.7
Monthly household income	Low	174	41.1
	Middle	184	43.5
	High	65	15.4
Current tobacco use	Yes	117	27.7
	No	306	72.3
Daily sugary food intake	Yes	176	41.6
	No	247	58.4
Brushes at least twice daily	Yes	193	45.6
	No	230	54.4
Regular preventive dental visits	Yes	136	32.2
	No	287	67.8

Distribution of OHL

The mean OHL score was 11.22 ± 3.55 out of 17 (median, 12; range, 1-17). Mean domain scores were 3.46 ± 1.58 for reading comprehension, 2.63 ± 1.12 for numeracy, 1.43 ± 0.69 for listening, and 3.70 ± 1.27 for decision-making. Low OHL was found in 131 participants (31.0%), moderate OHL in 66 (15.6%), and high OHL in 226 (53.4%). Domain scores and literacy classifications are presented in Table 2.

**Table 2:** Oral health literacy domain scores and literacy-level distribution. OHL: Oral health literacy. Values are presented as mean ± SD, median, and range for continuous variables and as n (%) for literacy categories. OHL categories: low, 0-9; moderate, 10-11; and high, 12-17.

Measure	Maximum score	Mean ± SD	Median	Score range	n	%
Reading comprehension	6	3.46 ± 1.58	4	0-6	-	-
Numeracy	4	2.63 ± 1.12	3	0-4	-	-
Listening	2	1.43 ± 0.69	2	0-2	-	-
Decision-making	5	3.70 ± 1.27	4	0-5	-	-
Total OHL score	17	11.22 ± 3.55	12	1-17	-	-
Low literacy	-	-	-	0-9	131	31
Moderate literacy	-	-	-	10-11	66	15.6
High literacy	-	-	-	12-17	226	53.4

Prevalence and severity of oral health outcomes

Dental caries was the most common condition (57.4%; 95% CI, 52.7-62.1). Tooth loss was recorded in 185 participants (43.7%; 95% CI, 39.1-48.5), and periodontal pocketing (highest sextant CPI ≥3) was observed in 175 (41.4%; 95% CI, 36.8-46.1). Poor oral hygiene was observed in 139 participants (32.9%; 95% CI, 28.6-37.5), and oral mucosal lesions were observed in 33 (7.8%; 95% CI, 5.6-10.8). The mean DMFT and OHI-S scores were 2.95 ± 2.01 and 2.61 ± 0.80, respectively. Prevalence and severity indicators are summarized in Table 3.

**Table 3:** Prevalence and severity of dental caries, periodontal pocketing, tooth loss, poor oral hygiene, and oral mucosal lesions. DMFT: Decayed, Missing, and Filled Teeth; OHI-S: Simplified Oral Hygiene Index. Prevalence is presented as n (%) with 95% CIs; continuous indicators are presented as mean ± SD.

Oral condition or indicator	n	Prevalence/mean	95% CI
Dental caries	243	57.40%	52.7-62.1
Periodontal pocketing (CPI ≥3)	175	41.40%	36.8-46.1
Tooth loss	185	43.70%	39.1-48.5
Poor oral hygiene	139	32.90%	28.6-37.5
Oral mucosal lesions	33	7.80%	5.6-10.8
DMFT score, mean ± SD	-	2.95 ± 2.01	-
OHI-S score, mean ± SD	-	2.61 ± 0.80	-

Association between OHL and oral health outcomes

The prevalence of the assessed oral health outcomes differed significantly across OHL categories. Dental caries was most frequent in the low-literacy group (71.0%), followed by the moderate-literacy (63.6%) and high-literacy (47.8%) groups (p < 0.001). Similar gradients were observed for periodontal pocketing (57.3%, 47.0%, and 30.5%), tooth loss (60.3%, 43.9%, and 34.1%), and poor oral hygiene (48.1%, 31.8%, and 24.3%) (all p < 0.001). Mean OHI-S scores also decreased significantly as literacy increased (p < 0.001). Differences in oral mucosal lesions and mean DMFT scores were not statistically significant. Comparisons across categories are presented in Table 4.

**Table 4:** Comparison of oral health indicators across oral health literacy categories. OHL: Oral health literacy; DMFT: Decayed, Missing, and Filled Teeth; OHI-S: Simplified Oral Hygiene Index. Values are presented as n (%) or mean ± SD, as appropriate. P-values were obtained using the chi-square test or Fisher’s exact test for categorical variables and one-way ANOVA for continuous variables, as appropriate.

Indicator	Low literacy (n = 131)	Moderate literacy (n = 66)	High literacy (n = 226)	p-value
Dental caries, n (%)	93 (71.0)	42 (63.6)	108 (47.8)	<0.001
Periodontal pocketing (CPI ≥3), n (%)	75 (57.3)	31 (47.0)	69 (30.5)	<0.001
Tooth loss, n (%)	79 (60.3)	29 (43.9)	77 (34.1)	<0.001
Poor oral hygiene, n (%)	63 (48.1)	21 (31.8)	55 (24.3)	<0.001
Oral mucosal lesions, n (%)	12 (9.2)	5 (7.6)	16 (7.1)	0.777
DMFT score, mean ± SD	3.12 ± 1.99	2.97 ± 2.06	2.84 ± 2.01	0.432
OHI-S score, mean ± SD	3.06 ± 0.91	2.62 ± 0.77	2.35 ± 0.76	<0.001

Factors associated with low-to-moderate OHL

In bivariate analysis, older age, rural residence, secondary education or below, tobacco use, brushing less than twice daily, and irregular preventive visits were associated with low to moderate OHL. In the adjusted model, rural residents had approximately twice the odds of low-to-moderate OHL compared with urban residents (adjusted OR, 1.97; 95% CI, 1.22-3.18; p = 0.006). Secondary education or below was the strongest predictor (adjusted OR, 5.89; 95% CI, 3.29-10.53; p < 0.001). Tobacco use, infrequent brushing, and irregular preventive visits were also independent predictors. Associations with age, sex, and low household income were not significant after adjustment. Full estimates are presented in Table 5.

**Table 5:** Bivariate and multivariable regression analyses of factors associated with low-to-moderate oral health literacy. Low-to-moderate oral health literacy was defined as an OHL-AQ score of 0-11. Adjusted odds ratios are from a single multivariable binary logistic regression model that included age, sex, residence, education, monthly household income, current tobacco use, toothbrushing frequency, and regular preventive dental visits simultaneously. Statistically significant values (p < 0.05) are those with 95% CIs excluding 1.0.

Factor	Crude OR (95% CI)	p-value	Adjusted OR (95% CI)	p-value
Age, per 1-year increase	1.03 (1.01-1.05)	0.01	1.02 (0.99-1.04)	0.06
Male sex	1.34 (0.88-2.03)	0.173	1.19 (0.74-1.93)	0.475
Rural residence	1.89 (1.25-2.87)	0.003	1.97 (1.22-3.18)	0.006
Secondary education or below	5.78 (3.34-10.00)	<0.001	5.89 (3.29-10.53)	<0.001
Low household income	1.51 (1.00-2.29)	0.052	1.35 (0.84-2.17)	0.221
Current tobacco use	1.97 (1.26-3.07)	0.003	1.88 (1.12-3.18)	0.018
Brushes less than twice daily	2.63 (1.70-4.08)	<0.001	2.19 (1.34-3.60)	0.002
No regular preventive dental visits	3.40 (2.02-5.74)	<0.001	3.23 (1.83-5.70)	<0.001

Sensitivity analysis excluding education from the multivariable model showed that the direction and magnitude of the associations were broadly similar to those in the primary analysis. Rural residence, current tobacco use, brushing less than twice daily, and the absence of regular preventive dental visits remained independently associated with low to moderate OHL, whereas age, male sex, and low household income were not statistically significant. This is shown in Appendix 1. Multicollinearity was assessed using variance inflation factors (VIFs) and tolerance values. VIFs ranged from 1.05 to 1.72, and tolerance values ranged from 0.581 to 0.952, indicating no evidence of problematic multicollinearity among the predictors (Appendix 2).

Independent association between OHL and oral health outcomes

After adjustment for age, sex, education, household income, and tobacco use, low OHL remained independently associated with a higher prevalence of several conditions. Compared with participants with high OHL, those with low OHL had a higher prevalence of dental caries (aPR, 1.46; 95% CI, 1.22-1.66), periodontal pocketing (CPI ≥3) (aPR, 1.73; 95% CI, 1.32-2.12), tooth loss (aPR, 1.75; 95% CI, 1.38-2.09), and poor oral hygiene (aPR, 1.80; 95% CI, 1.31-2.32). Moderate OHL was independently associated with dental caries and periodontal pocketing but not with tooth loss or poor oral hygiene. Adjusted estimates are presented in Table 6.

**Table 6:** Adjusted association of oral health literacy with dental caries, periodontal pocketing, tooth loss, and poor oral hygiene. PR: Prevalence ratio; CPI: Community Periodontal Index. Each outcome was modeled separately using modified Poisson regression with robust variance, adjusted for age, sex, education, monthly household income, and current tobacco use. High oral health literacy served as the reference category. Residence was not included because it was not prespecified as a confounder of the OHL-clinical outcome associations.

Outcome	Literacy comparison	Adjusted PR (95% CI)	p-value
Dental caries	Low vs high	1.46 (1.22-1.66)	<0.001
Dental caries	Moderate vs high	1.33 (1.03-1.58)	0.032
Periodontal pocketing (CPI ≥3)	Low vs high	1.73 (1.32-2.12)	<0.001
Periodontal pocketing (CPI ≥3)	Moderate vs high	1.50 (1.05-1.96)	0.03
Tooth loss	Low vs high	1.75 (1.38-2.09)	<0.001
Tooth loss	Moderate vs high	1.26 (0.86-1.70)	0.212
Poor oral hygiene	Low vs high	1.80 (1.31-2.32)	<0.001
Poor oral hygiene	Moderate vs high	1.25 (0.79-1.84)	0.32

## Discussion

This study found a consistent association between OHL and several oral health behaviors and clinically assessed oral health conditions among patients attending a tertiary dental hospital. Just over half of the participants (53.4%) had high OHL, whereas approximately one-third had low OHL, indicating substantial variation in patients’ ability to understand and use oral health information. Dental caries was the most frequently observed condition, followed by tooth loss and periodontal pocketing, consistent with the substantial global burden of dental caries and severe periodontal disease [5,6]. The observed gradient between lower OHL and less favorable oral health behaviors and clinical outcomes is also broadly consistent with previous evidence linking OHL with oral health behaviors and outcomes [3,4]. Among adult dental patients in India, Sukhabogi JR et al. similarly reported associations between OHL and oral health behaviors and outcomes [12].

In the crude analyses, increasing age, rural residence, lower educational attainment, tobacco use, less frequent toothbrushing, and absence of regular preventive dental visits were associated with low to moderate OHL. After adjustment for age, sex, residence, education, household income, tobacco use, toothbrushing frequency, and preventive dental visits, rural residence, lower educational attainment, tobacco use, less frequent toothbrushing, and absence of regular preventive dental visits remained independently associated with low to moderate OHL, whereas age, sex, and household income were no longer statistically significant. These findings suggest that educational attainment and related social and behavioral factors may play an important role in the distribution of OHL. Education may also capture differences in access to health information, socioeconomic circumstances, and health-related behaviors. Although the sensitivity analysis excluding education showed broadly similar directions and magnitudes of association, residual confounding related to education and socioeconomic circumstances cannot be excluded. The absence of problematic multicollinearity in the primary model, as indicated by the low VIF values, provides reassurance that the observed associations were not substantially affected by collinearity among the included predictors.

After multivariable adjustment, low OHL remained associated with a higher prevalence of dental caries, periodontal pocketing (CPI ≥3), tooth loss, and poor oral hygiene. Compared with participants with high OHL, those with low OHL had approximately 46%, 73%, 75%, and 80% higher prevalences of these conditions, respectively, based on the adjusted prevalence ratios. These findings are consistent with previous studies reporting associations between limited health or OHL and periodontal disease, dental plaque, oral health behaviors, and other oral health outcomes [4,8,14,15]. However, these adjusted associations should be interpreted as statistical associations rather than causal effects. Because the study was cross-sectional, the temporal direction cannot be established. Poor OHL may influence oral health behaviors and utilization, but existing oral health problems may also influence patients’ exposure to oral health information and their understanding of oral health needs. In addition, shared socioeconomic, behavioral, and healthcare-related factors may contribute to both low to moderate OHL and poorer oral health outcomes.

The association between limited OHL and tooth loss is consistent with previous population-based studies reporting relationships between health literacy, tooth loss, and the number of remaining teeth [16,17]. Tooth loss is a cumulative indicator of previous oral health experience and may therefore reflect long-term differences in preventive behaviors, dental attendance, access to care, and socioeconomic circumstances. In the present study, tooth loss was defined as the presence of at least one missing permanent tooth, and the analysis did not distinguish between disease-related and other causes of tooth loss. The observed association should therefore be interpreted cautiously and does not establish that low to moderate OHL directly contributed to tooth loss.

Behavioral and social factors, including rural residence, lower educational attainment, tobacco use, infrequent brushing, and irregular preventive dental attendance, were associated with low to moderate OHL. The association between rural residence and OHL is consistent with findings reported among rural adults [18]. The relationships involving brushing frequency and preventive dental attendance are particularly relevant because these factors may represent potential pathways through which OHL is related to oral health outcomes. However, mediation cannot be formally established in this cross-sectional study, and these variables should not be interpreted as demonstrated causal mediators. For patients with limited OHL, approaches such as plain-language counseling, visual instructions, teach-back communication, and simplified treatment or postoperative guidance may facilitate understanding and engagement with oral health care [1,3]. Digital health literacy may represent an additional consideration as oral health information increasingly becomes available through electronic platforms; previous research has reported associations between electronic health literacy and oral health outcomes [19].

The association between OHL and oral hygiene should also be interpreted cautiously. Although OHI-S scores differed across OHL categories, the findings are based on a clinical index that may be influenced by examiner-related measurement variability. In addition, the reported OHI-S values and classifications should be carefully reconciled across the Results, tables, and figures to ensure consistency before drawing definitive conclusions regarding this outcome. The lack of a formal intra-examiner reliability statistic further limits the certainty with which differences in clinical index measurements can be interpreted.

The broader relevance of oral health literacy should be considered within the established relationship between oral health and general health. Previous population studies have reported associations between poor oral health and mortality outcomes [20]. However, the present study did not assess systemic health outcomes and therefore cannot determine whether OHL or the observed oral health conditions have consequences for broader health status. Similarly, the findings should not be interpreted as demonstrating that improving OHL will necessarily reduce systemic disease or mortality. Rather, the results support the need for further longitudinal and interventional research examining whether improvements in OHL translate into sustained changes in oral health behaviors, healthcare utilization, and clinically assessed oral health outcomes.

From a public health perspective, the findings highlight the potential importance of addressing educational, socioeconomic, behavioral, and access-related factors alongside OHL. Oral health inequalities in India are influenced by multiple individual and structural determinants, including barriers to accessing dental services [21]. Community-based health literacy interventions in India remain relatively limited, and their effectiveness requires further evaluation [22]. Future studies should therefore examine whether interventions addressing OHL together with preventive service access and other socioeconomic barriers can improve clinically meaningful oral health outcomes.

Strengths

This study has several strengths. The use of the OHL-AQ, a previously developed multidomain instrument assessing reading comprehension, numeracy, listening, and decision-making, enabled the assessment of multiple dimensions of oral health literacy. In addition to self-reported information, the study incorporated clinically assessed oral health conditions using standardized diagnostic criteria, thereby providing an objective assessment of oral health status. Clinical examinations were performed by a trained examiner under standardized conditions using established clinical indices and criteria. The study also evaluated multiple oral health outcomes, including dental caries, periodontal pocketing, tooth loss, poor oral hygiene, and oral mucosal lesions, allowing a broader assessment of the relationship between OHL and oral health status. Multivariable regression analyses were performed to account for relevant sociodemographic and behavioral factors, and a sensitivity analysis excluding education was undertaken to assess the robustness of the findings.

Limitations

The cross-sectional design limits the ability to establish temporal or causal relationships between OHL, oral health behaviors, and clinically assessed oral health conditions. The observed associations should therefore not be interpreted as evidence that low to moderate OHL causes poorer oral health outcomes. The cross-sectional design also precluded formal assessment of potential mediation pathways. Variables such as brushing frequency, preventive dental visits, and dietary practices may represent behavioral pathways linking OHL with oral health outcomes; therefore, their associations should not be interpreted as evidence of independent causal effects.

The study was conducted at a single tertiary dental hospital, and the hospital-based sample may differ from individuals in the general community or those who do not seek dental care; consequently, the findings may have limited generalizability, and selection bias cannot be excluded. Several behavioral and healthcare-utilization variables, including tobacco use, brushing frequency, dietary practices, and preventive dental visits, were self-reported and may therefore be subject to recall and social desirability bias.

The OHL-AQ was administered in English, and although participants were considered sufficiently proficient to complete the questionnaire independently, no formal language or literacy proficiency assessment was performed; therefore, language-related measurement bias cannot be completely excluded. Although the clinical examiner underwent training and calibration, formal intra-examiner reliability statistics were not calculated, which may have introduced some measurement variability. The OHI-S findings should also be interpreted cautiously because index-based clinical measurements may be subject to measurement variability.

Potentially relevant clinical factors, including diabetes and glycaemic status, were not assessed and may have contributed to residual confounding. Education was strongly associated with low to moderate OHL and may overlap with socioeconomic and behavioral factors. Although multicollinearity was assessed using VIFs and a sensitivity analysis excluding education showed broadly similar findings, residual confounding related to education cannot be completely excluded. Oral mucosal lesions were identified clinically without routine histopathological confirmation, which may have resulted in misclassification of some lesions. In addition, only 33 participants had clinically identified mucosal lesions, limiting the statistical power and precision of association analyses for this outcome. Therefore, findings related to oral mucosal lesions should be interpreted cautiously. Because of the cross-sectional design, temporal relationships among OHL, behavioral factors, and clinical oral health outcomes could not be established. Therefore, behavioral variables such as toothbrushing frequency and preventive dental visits should not be interpreted as confirmed mediators or confounders solely on the basis of these data. Formal mediation analysis was not undertaken.

The sample size was determined primarily for estimating the prevalence of low-to-moderate OHL and was not specifically powered for each multivariable regression model involving multiple clinical outcomes and covariates. Consequently, the regression findings should be interpreted as exploratory/analytical associations, and the study may have had insufficient power to detect smaller associations. The definition of tooth loss as the presence of at least one missing permanent tooth may have captured relatively minor tooth loss and did not distinguish disease-related tooth loss from teeth lost for other reasons. Future multicenter studies using longitudinal designs, appropriately validated OHL instruments in relevant languages, formal examiner reliability assessment, and more detailed measures of tooth loss and relevant clinical conditions are warranted to further clarify these associations.

## Conclusions

Among patients attending a tertiary dental hospital, low OHL was independently associated with a greater burden of dental caries, periodontal pocketing, tooth loss, and poor oral hygiene, even after adjustment for sociodemographic and behavioral characteristics. Approximately one-third of participants had low OHL, and rural residence, lower educational attainment, tobacco use, and the absence of regular preventive visits were significantly associated with low-to-moderate OHL. These findings suggest that OHL assessment and literacy-sensitive patient education, such as plain-language communication, visual materials, teach-back methods, and community-based preventive programs, merit further evaluation in routine dental care. Because the study design was cross-sectional and no intervention was tested, the reported associations should be confirmed in prospective studies, and the effectiveness of literacy-based strategies should be established in controlled trials before firm causal or policy conclusions are drawn.

## Appendices

Appendix 1

**Table 7:** Sensitivity analysis of factors associated with low-to-moderate oral health literacy after excluding education from the multivariable model. Adjusted for age, sex, residence, household income, current tobacco use, brushing frequency, and regular preventive dental visits. Education was excluded from this sensitivity analysis.

Factor	Adjusted OR (95% CI)*	p-value
Age, per 1-year increase	1.02 (0.99-1.04)	0.065
Male sex	1.24 (0.79-1.98)	0.352
Rural residence	1.93 (1.20-3.10)	0.007
Low household income	1.45 (0.92-2.28)	0.108
Current tobacco use	1.91 (1.16-3.15)	0.012
Brushes less than twice daily	2.31 (1.45-3.68)	0.001
No regular preventive dental visits	3.30 (1.90-5.75)	<0.001

Appendix 2

**Table 8:** Variance inflation factors and tolerance values for predictors included in the multivariable model of low-to-moderate oral health literacy. VIF values below 5 were considered indicative of no problematic multicollinearity among the predictors included in the multivariable model. VIF: Variance inflation factor.

Variable	Variance Inflation Factor (VIF)	Tolerance (1/VIF)
Age (continuous)	1.12	0.893
Sex	1.05	0.952
Rural residence	1.48	0.676
Secondary education or below	1.72	0.581
Low household income	1.65	0.606
Current tobacco use	1.18	0.847
Brushes less than twice daily	1.25	0.8
No regular preventive dental visits	1.21	0.826
